# Glucagon stimulation test to assess growth hormone status in Prader–Willi syndrome

**DOI:** 10.1007/s40618-020-01367-6

**Published:** 2020-07-27

**Authors:** L. Casamitjana, O. Giménez-Palop, R. Corripio, R. Pareja, E. Berlanga, M. Rigla, JC. Oliva, A. Caixàs

**Affiliations:** 1 Endocrinology and Nutrition Department, Hospital Universitari Parc Taulí, C/ Parc Taulí, 1, 08208 Sabadell, Spain; 2 Medicine Department, Universitat Autònoma de Barcelona, Bellaterra, Spain; 3 Institut d’Investigació i Innovació Parc Taulí (I3PT), Sabadell, Spain; 4 Pediatrics Department, Hospital Universitari Parc Taulí, Sabadell, Spain; 5 Clinical Laboratory Department, UDIAT, Corporació Sanitària Universitària Parc Taulí, Sabadell, Spain; 6 Epidemiology Department, Fundació Parc Taulí, Sabadell, Spain

**Keywords:** Prader–Willi, Growth hormone deficiency, Glucagon-stimulation test, GHRH-arginine test

## Abstract

**Purpose:**

Growth hormone deficiency (GHD) must be confirmed before starting treatment in adults with Prader-Willi syndrome (PWS). Most studies use the growth-hormone-releasing hormone plus arginine (GHRH-arginine) test. No data are available on the glucagon stimulation test (GST) in PWS. We compared the utility of fixed-dose (1 mg) GST versus GHRH-arginine test in diagnosing GHD.

**Methods:**

Adults and late adolescents with PWS underwent both tests on separate days. In the GHRH-arginine test, GHD was defined according to body mass index. In the GST, two cutoffs were analyzed: peak GH concentration < 3 ng/mL and < 1 ng/mL. For analyses, patients were divided into two groups according to body weight (≤ 90 kg and > 90 kg).

**Results:**

We analyzed 34 patients: 22 weighing ≤ 90 kg and 12 weighing > 90 kg. In patients weighing ≤ 90 kg, the two tests were concordant in 16 (72.72%) patients (*k* = 0.476, *p* = 0.009 with GST cutoff < 3 ng/mL, and *k* = 0.450, *p* = 0.035 with GST cutoff < 1 ng/mL). In patients weighing > 90 kg, the two tests were not concordant with GST cutoff < 3 ng/mL, but were concordant in 11 (91.6%) patients (*k* = 0.833, *p* = 0.003) with GST cutoff < 1 ng/mL. GH peaks on the two tests correlated (*r* = 0.725, *p* = 0.008).

**Conclusion:**

Fixed-dose (1 mg) GST using a peak GH cutoff of < 3 ng/mL or < 1 ng/mL promises to be useful for screening for GHD in adults and late adolescents with PWS. However, in those weighing > 90 kg, the < 1 ng/mL cutoff seems better. Larger studies are necessary to establish definitive glucagon doses and cutoffs, especially in extremely obese patients.

## Introduction

Prader-Willi syndrome (PWS) is the most common syndromic form of obesity, occurring in approximately one in 10,000–30,000 live births, without sex differences in prevalence. PWS results from the loss of expression of paternal alleles in the PWS region of chromosome 15 [1]. PWS is characterized by hypotonia, high adiposity, low lean mass, hypogonadism, and growth hormone deficiency (GHD) [1, 2]. In children with PWS, it is widely accepted that treatment with growth hormone (GH) can be started without provocative tests to demonstrate GHD. GH treatment usually ends when the growth plates close and longitudinal bone growth finishes. However, after growth and body development, GHD can cause a wide variety of physical and psychological problems that can dramatically worsen quality of life. In early adolescence and adulthood, GH treatment can only be started if GHD is demonstrated by GH provocative tests [3].

Depending on the provocative tests and body mass index (BMI)-specific cutoffs used to evaluate GH status in different studies, the prevalence of GHD in PWS varies from 8 to 55% [4, 5]. To date, most studies that evaluated GH status in PWS used a standard growth-hormone-releasing hormone (GHRH) plus arginine (GHRH-arginine) test [6, 7] or, less frequently, an insulin tolerance test (ITT) [8]. The ITT has traditionally been accepted as the gold standard for assessing adult GHD, but it has potentially serious adverse effects and requires close medical supervision [9]. Furthermore, underlying insulin resistance can prevent normoglycemic and/or hyperglycemic obese patients from achieving the hypoglycemia necessary to stimulate GH secretion [10]. The GHRH-arginine test is considered the best alternative, but GHRH is expensive and not widely available in some countries. Moreover, GHRH-arginine might not be the optimal test for diagnosing GHD in PWS as GHRH stimulates both pituitary GH synthesis and release [11, 12], whereas arginine potentiates the stimulatory effects of GHRH by inhibiting hypothalamic somatostatin release [13]. These effects can lead to false-normal GH responses in patients with GHD of hypothalamic origin such as in PWS.

The glucagon stimulation test (GST) is an easy-to-perform, safe, inexpensive, and effective means of stimulating GH secretion with relatively few contraindications (e.g., pheochromocytoma or insulinoma) [14, 15]. Its mechanism of action is unclear, but it is apparently not influenced by hypothalamic deficiency [16]. However, glucagon’s effect on GH secretion seems to be weight-dependent [14], and most adults with PWS are obese. There is also no consensus on whether to use a fixed dose or a weight-based dose [17]. Some authors propose two different fixed glucagon doses depending on body weight (1 mg for patients weighing ≤ 90 kg and 1.5 mg for those weighing > 90 kg) [14, 16, 17] or on BMI (1 mg for patients with BMI < 30 kg/m^2^ and 1.5 for those with BMI ≥ 30 kg/m^2^) [15, 18], whereas others propose weight-based dosing (0.017 mg/kg [19] or 0.03 mg/kg body weight [17]). No prospective data are available about the use of the GST to evaluate GH status in patients with PWS. Thus, we aimed to compare the utility of a single fixed-dose (1 mg) GST versus the GHRH-arginine test for diagnosing GHD in adults and late adolescents with PWS.

## Materials and methods

## Patients

We included all adult and late adolescent patients with PWS treated at our center between January 1, 2016 and January 31, 2018 (*N* = 34).

All subjects underwent cytogenetic analysis. Height was determined by a Harpenden Stadiometer (Holtain Ltd, Dyfed, UK). Body weight was measured to the nearest 0.1 kg with standard equipment. Body mass index (BMI) was defined as weight in kilograms divided by the square of height in meters. According to the World Health Organization classification [20], normal weight was defined as BMI 18.5–24.9 kg/m^2^, overweight as BMI 25–29.9 kg/m^2^, and obese as BMI $\ge$ 30 kg/m^2^.

## Endocrine protocol

All subjects underwent a standard GHRH-arginine test and a standard GST on separate days ranging from 8 to 15 days apart, being randomly assigned to start with one test or the other with a random–number generator [21].

Both tests started at 8 AM after overnight fasting and were done with patients in a recumbent position.

## GHRH-arginine test

A catheter was placed in the antecubital vein; 15 min later 1 µg/kg GHRH_1-29_(GHRH, Ferring GmbH, Kiel, Germany) was injected as a bolus. In the 30 min after GHRH administration, 0.5 g/kg (maximum dose 30 g) L-arginine hydrochloride (Torbay Pharmaceuticals, Paignton, Devon, UK) diluted in 300 cc of saline 0.9% sodium chloride was infused. Blood samples for GH determination were drawn at -15, 0, 30, 45, 60, 90, and 120 min after the intravenous bolus of GHRH. GH deficiency was defined as a peak concentration < 11 ng/mL if BMI < 25 kg/m^2^, < 8 ng/mL if BMI 25–30 kg/m^2^, and < 4 ng/mL if BMI ≥ 30 kg/m^2^ [22].

## GST

All subjects received an intramuscular injection of 1 mg of glucagon (Novo-Nordisk, Bagsvaerd, Denmark) in the deltoid muscle. Blood samples for glucose and GH determinations were drawn every 30 min from baseline until 180 min [14]. GHD was defined in two ways: according to the classical definition (peak concentration < 3 ng/mL) [14, 15] and according to the definition recently proposed by Hamrahian et al. [17] (peak concentration < 1 ng/mL).

GH concentrations were measured by chemiluminescent immunoassay (LIAISON hGH, Diasorin S.p.A, Saluggia, Italy). The sensitivity was 0.05 ng/mL and intra-assay coefficients of variation (CV) were 2.26% at 3.48 ng/mL and 1.93% at 17.3 ng/mL.

## Statistical analyses

Continuous variables are reported as medians and interquartile ranges (IQR). Categorical variables are reported as frequencies and percentages. To assess the concordance between diagnostic tests, we used the Kappa index and its standard error. To compare GH peak concentration between the two tests, we used the Mann -Whitney U test and for multiple comparisons we used the Kruskal–Wallis test. To study the relationship between variables, we used regression analysis. Statistical significance was fixed at *p* < 0.05. Because using a single 1 mg dose of glucagon in patients weighing > 90 kg is not widely accepted, for the purpose of analysis we divided patients into two groups according to body weight (≤ 90 kg and > 90 kg). All analyses were done with IBM SPSS Statistics for Windows, version 25.0 (IBM Corp., Armonk, N.Y., USA).

## Results

### Patients weighing ≤ 90 kg

We evaluated 22 patients with PWS who weighed ≤ 90 kg [18 adults and 4 late adolescents; 5 male and 17 female; median age, 23.5 y (IQR: 19.7–36.2), range 15–47 y; median BMI, 30.7 kg/m^2^ (IQR: 24.8–34.0)]. Of these, 5 were normal weight, 5 overweight, and 12 obese, including 1 with BMI ≥ 40 kg/m^2^. Cytogenetic analysis revealed that 14 had microdeletions (6 type I, 8 type II), 5 had maternal uniparental disomy, and 3 had imprinting defects. Prior to the study, 15 patients had undergone GH therapy; all had stopped the therapy at least 1 year before enrollment. At the time of the study, 10 patients were undergoing sex steroid replacement therapy. A total of 5 patients had type 2 diabetes (2 treated only with oral agents, 2 with oral agents and GLP-1 analogues, and 1 with oral agents and insulin); metabolic control was acceptable (glycosylated hemoglobin < 7%). Table 1 reports the characteristics of each patient, peak GH concentrations in the two tests, and whether GHD was diagnosed according to each test and cutoff.

**Table 1:** Characteristics of patients weighing ≤ 90 kg

	Sex (M/F)	Age( years)	Weight (kg)	BMI (kg/m^2^)	Genetic subtype	GH treatment in childhood	Peak GH GHRH-arginine (ng/mL)	GHD (GHRH- arginine)	Peak GH GST (ng/mL)	GHD (GH peak < 3 ng/mL) in GST	GHD (GH peak < 1 ng/mL) in GST
1	M	17	73.1	23.3	D II	Yes	13.7	No	14.4	No	No
2	M	38	73	28.9	MUD	Yes	3.58	Yes	1.38	Yes	No
3	M	39	79.5	29.9	ID	No	1.52	Yes	0.11	Yes	Yes
4	M	28	53.6	32.4	MUD	No	1.2	Yes	0.18	Yes	Yes
5	M	47	84	34.3	D II	No	0.42	Yes	0.99	Yes	Yes
6	F	15	51	23.3	MUD	Yes	14.7	No	12.3	No	No
7	F	16	60	23.8	MUD	Yes	25.8	No	1.24	Yes	No
8	F	23	54.4	23.9	D I	Yes	55	No	12	No	No
9	F	24	65	24.2	ID	Yes	12.5	No	0.1	Yes	Yes
10	F	23	55	25.1	D I	Yes	50.3	No	1.32	Yes	No
11	F	22	74.6	27.1	D II	Yes	6.38	Yes	2.57	Yes	No
12	F	37	72	28.3	D II	No	5.08	Yes	1.06	Yes	No
13	F	33	65	30.5	D I	Yes	50.5	No	3.77	No	No
14	F	23	70.7	31	D I	Yes	8.05	No	0.57	Yes	Yes
15	F	17	72	31.2	D II	Yes	11.4	No	4.57	No	No
16	F	23	80	32.8	MUD	Yes	3.02	Yes	0.22	Yes	Yes
17	F	30	82.5	32.9	ID	Yes	3.13	Yes	0.05	Yes	Yes
18	F	19	89	33.9	D II	Yes	12.5	No	0.6	Yes	Yes
19	F	36	68.5	34.5	D II	No	2.67	Yes	0.25	Yes	Yes
20	F	42	77.6	35.4	D II	No	6.13	No	7.81	No	NO
21	F	20	84.4	37.5	D I	No	10.8	No	1.26	Yes	No
22	F	24	87	43.1	D I	Yes	2.67	Yes	0.32	Yes	Yes

*BMI* body mass index, *GH* growth hormone, *GHD* growth hormone deficiency, *GHRH-arginine* growth-hormone-releasing hormone plus arginine, *GST* glucagon stimulation test, *D I* type I deletion, *D II* type II deletion, *MUD* maternal uniparental disomy, *ID* imprinting defect

#### GHRH-arginine test

According to the established BMI-related cutoffs, 10 (45.5%) patients met the criteria for GHD. Of these 10 patients, 4 were overweight and 6 obese; of the 12 that did not meet the criteria for GHD, 5 were normal weight, 1 overweight, and 6 obese. No adverse effects occurred during the test. Median peak GH concentration was 8.19 ng/mL (IQR: 3.1–14.0 ng/mL).

#### Glucagon-stimulation test

According to the classical cutoff (peak concentration < 3 ng/mL), 16 (72.7%) patients met the criterion for GHD. Of these 16 patients, 2 were normal weight, 5 overweight, and 9 obese; of the 6 that did not meet the criterion for GHD, 3 were normal weight and 3 obese. The GST classified all patients weighing > 79.5 kg (*n* = 6) as GHD.

According to the recently proposed cutoff (1 ng/mL), 10 (45.5%) met the criterion for GHD. Of these 10 patients, 1 was normal weight, 1 overweight, and 8 obese; of the 12 that did not meet the criterion for GHD, 4 were normal weight, 4 overweight, and 4 obese.

No adverse effects occurred during the test. In 18 (81.8%) patients, the concentration of GH peaked between 120 and 180 min after glucagon administration. Median peak GH concentration was 1.15 ng/mL (IQR: 0.24–3.97 ng/mL).

#### Concordance between the GST and the GHRH-arginine test

Using the < 3 ng/mL cutoff for GST, the two tests were concordant in 16 (72.7%) patients and discordant in 6 (27.3%) (*k* = 0.476, *p* = 0.009) (Table 2). Using the < 1 ng/mL cutoff, the two tests were concordant in 16 (72.7%) patients and discordant in 6 (27.3%) (*k* = 0.450, *p* = 0.035) (Table 3).

**Table 2:** Contingency table in patients weighing ≤ 90 kg (*n* = 22). growth hormone deficiency (GHD) according to the glucagon stimulation test (GST) using peak growth hormone concentration < 3 ng/mL as the cutoff versus according to the growth-hormone-releasing hormone (GHRH)-arginine test

GHD with GST (GH peak < 3 ng/mL), *n* (%)	GHD with the GHRH-arginine test *n* (%)	
	No	Yes
No	6 (100.0%)	0 (0.0%)
Yes	6 (37.5%)	10 (45.5%)

**Table 3:** Contingency table in patients weighing ≤ 90 kg (*n* = 22). growth hormone deficiency (GHD) according to the glucagon stimulation test (GST) using peak growth hormone concentration < 1 ng/mL as the cutoff versus according to the growth-hormone-releasing hormone (GHRH)-arginine test

GHD with GST (GH peak < 1 ng/mL), *n* (%)	GHD with the GHRH-arginine test *n* (%)	
	No	Yes
No	9 (75.0%)	3 (25.0%)
Yes	3 (30.0%)	7 (70.0%)

#### Subgroup analyses

In the 15 patients that underwent GH treatment during childhood, peak GH concentration on the GHRH-arginine test was higher than in those who did not receive GH during childhood [12.5 ng/mL (IQR: 3.58–25.8 ng/mL) vs. 3.10 ng/mL (IQR: 1.52–6.13 ng/mL), respectively, *p* = 0.009]. On the GST, GH peaks were similar: [1.32 ng/mL (IQR: 0.32–4.57 ng/mL) for patients with GH treatment in childhood vs. 1.00 ng/mL (IQR: 0.18–1.26 ng/mL) for those without, *p* = 0.332]. Those who received GH treatment during childhood were younger [23.0 years (IQR: 19.6–26.7 years) vs. 37.0 years (IQR: 27.3–43.9 years), respectively, *p* = 0.007] and had lower BMI [28.9 kg/m^2^ (IQR: 25.9–32.0 kg/m^2^) vs. 34.3 kg/m^2^ (IQR: 30.2–36.2 kg/m^2^), respectively, *p* = 0.039].

Regarding the genetic subtype, those with imprinting defects had the lowest peak concentrations of GH on both tests, being significant only for the GST [Deletion type I: 1.29 ng/mL (IQR: 0.51–5.83 ng/mL), Deletion type II: 1.82 ng/mL (IQR: 0.70–7.00 ng/mL), Imprinting defect: 0.10 ng/mL (IQR:0.05-not calculable ng/mL), Maternal uniparental disomy: 1.24 ng/mL (IQR: 0.20–6.84 ng/mL), p = 0.048]. This significance did not disappear after adjustment for age, weight, or BMI.

Peak GH was higher in females than in males on the GHRH-arginine test [10.8 ng/mL (IQR: 4.11–20.3 ng/mL) vs. 2.10 ng/mL (IQR: 1.36–8.64 ng/mL), respectively, *p* = 0.039)], but not on the GST [1.24 ng/mL (IQR: 0.29–4.17 ng/mL) in females vs. 1.0 ng/mL (IQR: 0.15–7.89 ng/mL) in males, *p* = 0.820)].

There were no differences in GH response on either test between patients treated with sex steroids versus those not treated with sex steroids in the whole group or separated by sex (data not shown).

#### Regression analysis

**Fig. 1:**
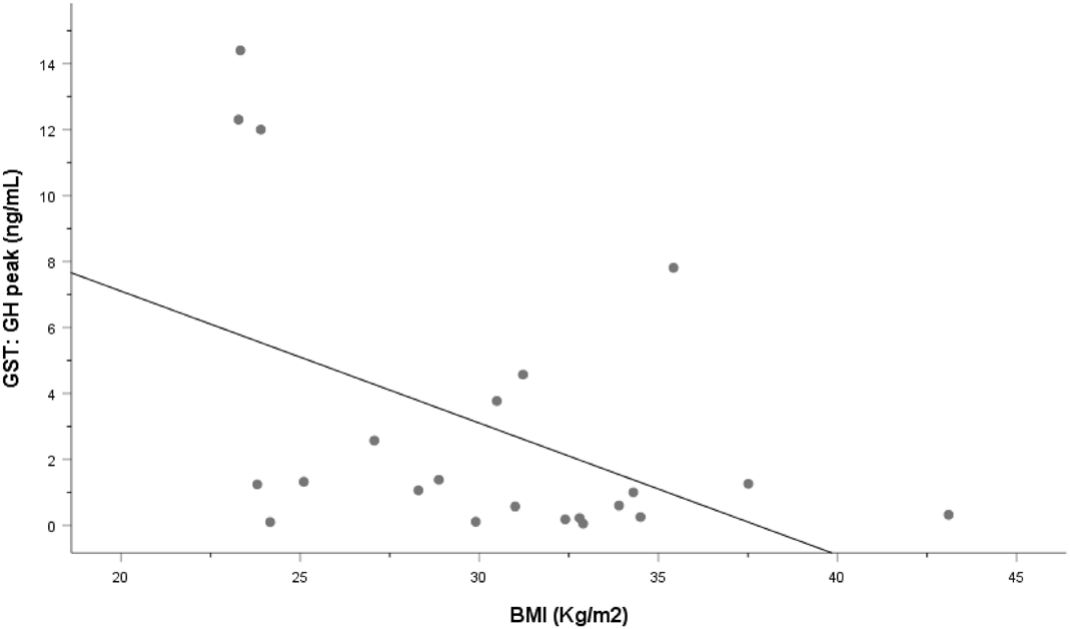
Correlation between peak growth hormone (GH) concentration and body mass index (BMI) on the glucagon-stimulation test (GST) in patients weighing ≤ 90 kg. Spearman correlation test, *r* =− 0.469, *p* = 0.028

Peak concentrations of GH observed on the two tests did not correlate (*r* = 0.330, *p* = 0.134). GH peak on the GHRH-arginine test correlated negatively with BMI (*r* = − 0.465, *p* = 0.029) and weight (*r* = − 0.562, *p* = 0.06), but not with age. Peak GH on the GST correlated negatively with BMI (*r* = − 0.469, *p* = 0.028) (Fig. 1), but not with age (*r* = 0.293, *p* = 0.186) or with weight (*r* = 0.368, *p* = 0.092).

### Patients weighing > 90 kg

We evaluated 12 patients with PWS who weighed > 90 kg [12 adults; 7 male and 5 female; median age, 26 y (IQR: 19.0–32.3), range 18–51 y; median BMI, 41.0 kg/m^2^ (IQR: 34.9–49.2)]. All were obese, 7 with BMI ≥ 40 kg/m^2^. Cytogenetic analysis revealed that 10 had microdeletions (5 type I, 4 type II, and 1 atypical BP2-BP4 microdeletion) and 2 had maternal uniparental disomy. Prior to the study, 4 patients had undergone GH therapy; all had stopped the therapy at least 1 year before enrollment. At the time of the study, 8 patients were undergoing sex steroid replacement therapy. Two patients had type 2 diabetes (1 treated with oral agents, GLP-1 analogues, and insulin, and the other with oral agents and insulin); metabolic control was acceptable (glycosylated hemoglobin < 7%). Table 4 reports the characteristics of each patient, peak GH concentrations in the two tests, and whether GHD was diagnosed according to each test and cutoff.

**Table 4:** Characteristics of patients weighing more than 90 kg

	Sex (F/M)	Age (years)	Weight(kg)	BMI (kg/m^2^)	Genetic subtype	GH treatment in childhood	Peak GH GHRH-arginine (ng/mL)	GHD with GHRH -arginine	Peak GH GST (ng/mL)	GHD with GST ( peak < 3 ng/mL)	GHD with GST ( peak < 1 ng/mL)
1	M	51	93.8	30.6	D I	No	1.78	Yes	0.14	Yes	Yes
2	M	19	110	34.8	D II	Yes	1.25	Yes	0.14	Yes	Yes
3	M	18	127.2	34.8	D I	Yes	6.22	No	1.53	Yes	No
4	M	19	98.4	35.3	AD	No	6.55	No	1.57	Yes	NO
5	M	30	104	40.1	D I	No	1.48	Yes	0.05	Yes	Yes
6	M	30	103.5	44.2	D I	No	4.17	No	1.48	Yes	No
7	M	33	109	54	D II	No	3.31	Yes	0.09	Yes	Yes
8	F	22	90.5	35	MUD	Yes	8.84	No	1.18	Yes	No
9	F	39	91.5	41.8	D II	No	2.48	Yes	1.19	Yes	No
10	F	19	129.5	47.5	D II	Yes	10.6	No	1.24	Yes	No
11	F	28	115	49.7	D II	No	0.78	Yes	0.27	Yes	Yes
12	F	24	125	62.9	MUD	No	1.34	Yes	0.05	Yes	Yes

*BMI* body mass index, *GH* growth hormone, *GHRH-arginine* growth-hormone-releasing hormone plus arginine, *GHD* GH deficiency, *GST* glucagon stimulation test, *D I* type I deletion, *D II* type II deletion, *AD* atypical microdeletion, *MUD* maternal uniparental disomy

#### GHRH-arginine test

According to the established BMI-related cutoffs, 7 (58.3%) patients met the criteria for GHD. Median peak GH concentration was 2.90 ng/mL (IQR: 1.38–6.47 ng/mL).

#### Glucagon-stimulation test

According to the classical cutoff (peak concentration < 3 ng/mL), all patients, met the criterion for GHD.

According to the recently proposed cutoff (peak concentration < 1 ng/mL), 6 (50%) met the criterion for GHD. Of these 6 patients, 4 had BMI > 40 kg/m^2^.

No adverse effects occurred during the test. In 10 (83.3%) patients, the concentration of GH peaked between 120 and 180 min after glucagon administration. Median peak GH concentration was 0.73 ng/mL (IQR: 0.10–1.42 ng/mL).

#### Concordance between the GST and the GHRH-arginine test

Using the < 3 ng/mL cutoff, because all patients met the criterion for GHD on the GST, the two tests were not concordant (*k* = 0).

Using the < 1 ng/mL cutoff, the two tests were concordant in 11 (91.7%) patients (*k* = 0.833, *p* = 0.003) (Table 5).

**Table 5:** Contingency table in patients weighing > 90 kg (*n* = 12): growth hormone deficiency (GHD) according to the glucagon stimulation test (GST) using peak growth hormone concentration < 1 ng/mL as the cutoff versus according to the growth-hormone-releasing hormone (GHRH)-arginine test

GHD with GST ( GH peak < 1 ng/mL)	GHD with the GHRH-arginine test *n* (%)	
	No	Yes
No	5 (83.3%)	1(16.7%)
Yes	0 (0.0%)	6 (100.0%)

#### Regression analysis

**Fig. 2:**
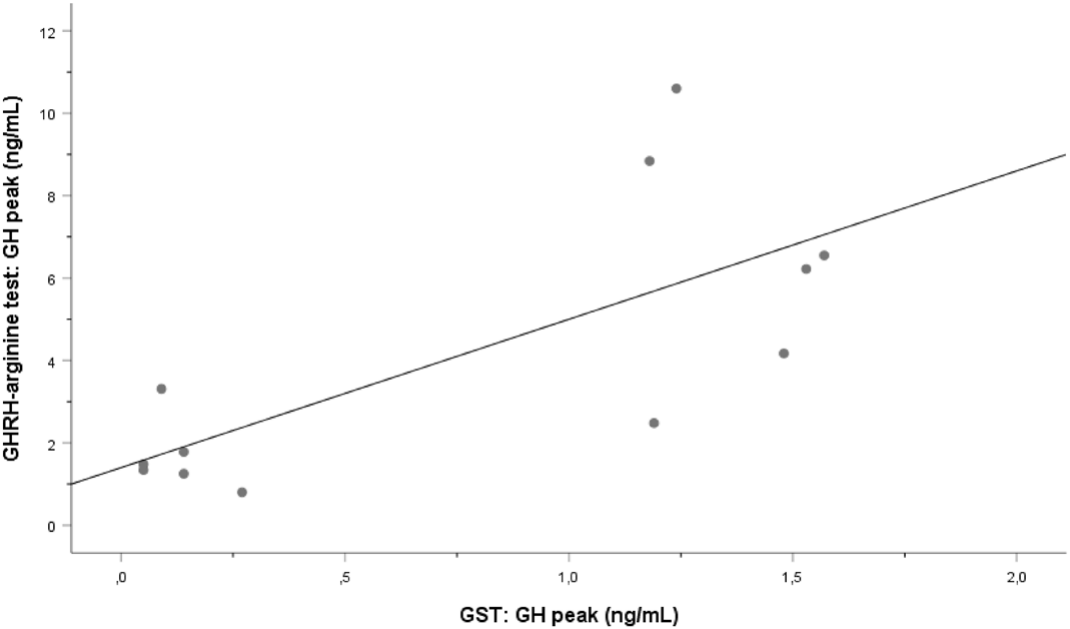
Correlation between peak growth hormone (GH) concentration on the growth-hormone-releasing hormone (GHRH)-arginine test and on the glucagon-stimulation test (GST) in patients weighing > 90 kg. Spearman correlation test, *r* = 0.725, *p* = 0.008

There was a good correlation between peak GH concentrations on the two tests (*r* = 0.725, *p* = 0.008) (Fig. 2). No correlations were observed between peak GH and BMI, weight, or age on either test.

## Discussion

We aimed to evaluate the utility of a GST using a fixed-dose (1 mg) of glucagon administered intramuscularly in diagnosing GHD in adults and late adolescents with PWS by comparing the results with those achieved with the GHRH-arginine test. In patients weighing ≤ 90 kg, the GST diagnosis was concordant with the GHRH-arginine test diagnosis, regardless of whether the peak GH cutoff was < 3 ng/mL or < 1 ng/mL. However, in patients weighing > 90 kg, only the < 1 ng/mL peak GH cutoff had good concordance with the GHRH-arginine test. These findings suggest that GST could be used in cases where other, more sophisticated tests such as ITT or GHRH-arginine test might be complicated to perform because they require intravenous access (sometimes difficult in patients with PWS) and have other drawbacks. Moreover, the GHRH-arginine test might not be the best test for diagnosing GHD in PWS as it stimulates mainly the pituitary secretion and can lead to false-negative diagnoses for GHD in hypothalamic diseases such as PWS.

The GST does not require intravenous access, is reproducible, safe, and inexpensive; moreover, it is not influenced by sex or hypothalamic GHD [16]. GST has mild adverse effects including nausea, vomiting, and headaches; more rarely and mostly in elderly patients, GST can cause hypotension, hypoglycemia, or seizures [23].

Most [9, 14, 17] but not all [15] authors propose a fixed intramuscular dose of 1 mg glucagon for patients weighing < 90 kg and 1.5 mg for those weighing ≥ 90 kg. Moreover, some authors have suggested that using these fixed doses and a peak GH concentration cutoff < 3 ng/ml as recommended in the 2009 update of the American Association of Clinical Endocrinologists guidelines [24] may potentially overdiagnose adult GHD in many overweight/obese subjects and in those with glucose intolerance [25]. For this reason, Hamrahian et al. [17] suggested changing the peak GH cutoff to < 1 ng/mL for the same glucagon fixed doses mentioned above and < 2 ng/mL when glucagon was used in a weight-based dose (0.03 mg/kg). These authors also concluded that the 3 ng/mL cutoff GH peak misdiagnosed some GH-sufficient adults.

In the present study, we used a fixed dose of 1 mg of glucagon for all patients, and we analyzed those weighing ≤ 90 kg and those weighing > 90 kg separately.

In the group weighing ≤ 90 kg, peak GH concentrations on GST correlated negatively with BMI. These results are in accordance with those of a previous study where peak GH concentrations correlated negatively with weight and BMI in healthy controls [14]. Other studies also found that the negative correlation between BMI and peak GH concentrations in the GST was stronger in patients with BMI between 30 kg/m^2^ and 40 kg/m^2^ and seemed to plateau for those with BMI > 40 kg/m^2^ [25, 26]. In our cohort, all subjects weighing > 79.5 kg (*n* = 18) failed to achieve peak GH concentrations > 3 ng/mL in the GST. However, in those weighing > 90 kg, defining GHD as a peak GH concentration < 1 ng/mL yielded results more concordant with those of the GHRH-arginine test and peak GH in the two tests showed a good correlation.

In the analysis of subgroups of the patients weighing ≤ 90 kg, patients who received GH treatment during childhood had a higher GH peak on the GHRH-arginine test, probably because this group was younger and had lower BMI than the group not treated with GH during childhood.

The lowest GH peak found in the imprinting defect genetic subtype, even after adjusting for age and BMI, must be interpreted with caution given the lack of power due to the sample size. However, other authors have also reported that the pattern of GH secretion varied by genetic subtype, with higher GH responses in typical deletion subjects than in patients with disomy [27]. Further studies are needed to elucidate the significance of this finding.

The higher GH peak on the GHRH-arginine test in females than in males could be related to an estrogenic effect. Several earlier studies have shown that estrogens can enhance the GH response to most stimuli, such as hypoglycemia and arginine, in men and in premenopausal and postmenopausal women [28]. By contrast, no sex-based difference in GH response to glucagon was observed in this study or in others [29, 30], and exogenous estrogen administration is ineffective in enhancing the GH response to glucagon in men [29].

The mechanisms by which glucagon stimulates GH secretion remain unclear [9]. It seems that peak GH concentrations do not directly depend on glycemic levels [31]. One possible mechanism could be that breakdown of glucagon in the muscles produces a peptidyl fragment that promotes the release of GH [19]. Additionally, glucagon induces norepinephrine secretion, which might stimulate the release of GH via α-receptors [16]; this mechanism could be of great interest in PWS because patients with PWS have underlying autonomic dysfunction [32].

The route of glucagon administration may also be important in the release of GH. Intravenous glucagon causes less GH release than both intramuscular and subcutaneous glucagon [33]. We chose the intramuscular route because it has been suggested to be more reliable and effective than the subcutaneous route [30]. However, new routes, such as intranasal glucagon, recently approved by FDA [34] to treat hypoglycemia, might also prove effective, although to our knowledge there is insufficient information about its effectiveness in provoking the release of GH.

- Our study has several limitations. First, we compared the results of the GST against GHRH-arginine test rather than against the gold standard (ITT). However, the ITT has serious drawbacks in PWS patients, and the GHRH-arginine test is more widely used. Second, our analyses may have been underpowered to detect small effects, given the relatively small sample size including patients with a wide range of BMI. The number of patients weighing > 90 kg was low (*n* = 12), and they received 1 mg glucagon rather than the 1.5 mg recommended by other authors. Nevertheless, using the cutoff < 1 ng/mL, we obtained good concordance between the two tests in this group of patients. Finally, we did not take glycemic status into account. Whether glucose levels can interfere with the response in the GST is controversial. Higher blood glucose levels (whether fasting or peak and nadir) during the GST have been associated with lower peak GH responses in some studies [25, 35]; however, other authors consider that blood glucose levels do not impair glucagon-induced GH release [29, 36].

In conclusion, a fixed-dose (1 mg) GST using a peak GH cutoff of < 3 ng/mL or < 1 ng/mL seems useful for screening for GHD in adults and late adolescents with PWS. However, in those weighing > 90 kg, the < 1 ng/mL cutoff is more suitable. Larger studies are necessary to corroborate our findings and establish definitive glucagon doses and cutoffs, especially in patients with extreme obesity or glucose intolerance.
